# A Validated Adsorptive Stripping Voltammetric Determination of Antidiabetic Agent Pioglitazone HCl in Tablets and Biological Fluids

**Published:** 2008-12

**Authors:** Nawal Ahmad Al-Arfaj, Eman Abdullah Al-Abdulkareem, Fatma Ahmad Aly

**Affiliations:** *Department of Chemistry, College of Science, Women Student-Medical Studies and Science Sections, King Saud University, Saudi Arabia*

**Keywords:** pioglitazone HCl, square-wave, adsorptive stripping voltammetry, biological fluids, actos tablets, pharmacokinetic

## Abstract

Square-wave adsorptive cathodic stripping voltammetry was used to determine pioglitazone HCl in Britton Robinson buffer of pH5. The adsorptive cathodic peak was observed at -1.5 V vs. Ag/AgCl. The peak response was characterized with respect to pH, supporting electrolyte, frequency, scan increment, pulse-amplitude, accumulation potential and pre-concentration time. Under optimal conditions, the peak current is proportional to the concentration of pioglitazone HCl, and a linear calibration graphs were obtained within the concentration levels of 10^-8^ and 10^-4^ M following different accumulation time periods (0-300 s). The obtained results were analyzed and the statistical parameters were calculated. The detection limit is 8.08 × 10^-9^ M (3.17 ng ml^-1^) using 300 s pre-concentration time, whereas the quantitative limit is 2.45 × 10^-8^ M (9.63 ng ml^-1^). The proposed method was applied to assay the drug in pharmaceutical formulations and biological fluids. The pharmacokinetic parameters of drug in human plasma were estimated as: *C*_max_=785.8 ng ml^-1^, *t*_max_=1.5 h, *K*_e_=0.125 h^-1^ and *t*_1/2_=8 h which are favorably compared with those reported in literature.

## INTRODUCTION

**Figure 1:**
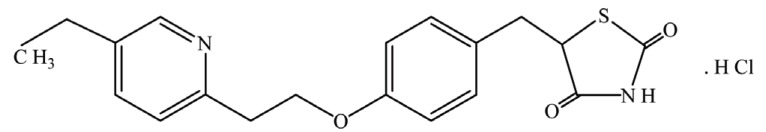
Structure of pioglitazone HCl.

Pioglitazone hydrochloride, (±)-5-{4-[2-(5-ethyl-2-pyridyl)ethoxy]benzyl}-2,4-thiazolidinedione hydrochloride salt (Fig. 1), is an oral antidiabetic agent that has been shown to affect abnormal glucose and lipid metabolism associated with insulin resistance by enhancing insulin action on peripheral tissues in animal models (1, 2). It is used in the treatment of type-II diabetes (non-insulin-dependent diabetes mellitus, NIDDM also known as adult-onset diabetes) (3, 4).

Several analytical methods have been reported for the determination of pioglitazone HCl in bulk form, pharmaceuticals and biological fluids. Most of the reported methods are chromatographic, and no official methods have been reported for the determination of pioglitazone HCl. The reported methods include: high performance liquid chromatography with ultraviolet detection (HPLC-UV) (5-15), high performance liquid chromatography with mass spectroscopy (HPLC/MS) (16), high performance liquid chromatography with tandem mass spectroscopy (HPLC/MS/MS) (17-19), high performance thin layer chromatography (HPTLC) (20, 21), thin layer chromatography (TLC) (22, 23), capillary electrophoresis (CE) (24, 25), and miceller electrokinetic chromatography (MEKC) (26). Other reported methods include a potentiometric method (27) and UV spectrophotometric methods (28, 29). To date no voltammetric procedure has been reported for the assay of pioglitazone HCl.

Adsorptive stripping voltammetric analysis, especially with the square-wave waveform, is an extremely simple and sensitive technique that can be used for analysis of drugs without the requirement for extraction steps prior to the assay. Moreover, square-wave voltammetry is a large-amplitude differential technique in which a waveform composed of a symmetrical square wave is applied to the working electrode (30). The current is sampled twice during each square-wave cycle, once at the end of the forward pulse and once at the end of the reverse pulse. The resulting peak current is proportional to the concentration of the analyte. Excellent sensitivity accrues from the fact that the net current is larger than either the forward or the reverse components current. Coupled with the effective discrimination against the charging current, very low detection limits can be attained. Furthermore, stripping voltammetry is an important technique for trace determination of many inorganic and organic substances (31). The stripping technique has been used successfully for the determination of several drugs (32-38).

The aim of this work is the quantitative determination of pioglitazone HCl using square-wave adsorptive cathodic stripping voltammetry (SWAdCS). This method is simple, rapid, sensitive and easy to apply in pharmaceutical formulations and in biological samples.

## EXPERIMENTAL

### Apparatus

Electrochemical Analyzer (746 VA Trace Analyzer-Metrohm/Switzerland) was used for SWAdCS voltammetric measurements and Electrochemical Analyzers (797 VA Cumputrace Analyzer-Metrohm/Switzerland) was used for cyclic voltammetry. The electrode assembly of a hanging mercury drop electrode (HMDE) as a working electrode (area=0.05 cm^2^) for SWAdCS and cyclic voltammetries. Ag/AgCl/KCl_sat_. was the reference electrode and a platinum wire supplied as auxiliary electrode, was used. Stirring of the solution in the electrolysis cell was performed using a complete stirrer comprised as a part of the electrochemical analyzers to provide convective transport during the preconcentration step. The measurements were semi-automated and controlled through the programming capacity of the apparatus. The data were recorded using an external printer attached to the two RS232 interfaces of the 746 VA Trace Analyzer.

A UV-Vis Spectrophotometer Model (Ultrospec 2100-Pro) was used for a comparative assay of drug in tablets by means of a published spectrophotometric method (28), and for the spectral study of pioglitazone HCl.

A (Hann pH211-Romania) pH-meter with combined glass and calomel (saturated KCl) electrodes was used for the pH measurements of the supporting electrolytes.

The de-ionized water used throughout the present study was supplied from (Elgastat Micromeg-England).

### Materials and reagents

**Materials.** Pure drug sample of pioglitazone HCl was used (Chargen-Zert.Brand, Germany); Tablets containing pioglitazone HCl (Takeda chemical industries Ltd., Osaka. Japan), were purchased from commercial sources, serum samples were supplied from (United Diagnostics Industry K.S.A.), urine samples were obtained from healthy volunteers. Human plasma samples were provided by (King Khalid University Hospital, Riyadh, Saudi Arabia).

**Reagents.** The following reagents were used: Britton-Robinson (B-R) buffers (0.08 M) (39), of pH2-11, acetate buffer (39), of pH4.5-5.5, sodium sulphate (Fluka, Switzerland) 0.1 M; sodium nitrate (Fluka, Switzerland) 0.1 M; sodium perchlorate (Fluka, Switzerland) 0.1 M and potassium chloride (Fluka, Switzerland) 0.1 M, were prepared using de-ionized water (40), and used as the supporting electrolytes; disodium tetraborate (BDH Ltd., UK) 0.02 M; dichloromethane (BDH Ltd., UK), and methanol (BDH Ltd., UK) were used.

### Procedure

**General analytical procedure.** Stock solution (1 × 10^-3^ M) of pioglitazone HCl was prepared in methanol. This solution was further diluted with methanol to give the appropriate concentrations of working standard solutions. Aliquots of these solutions in the concentration range cited in (Table 1) were transferred into a series of 25 ml volumetric flasks and diluted to the mark with B-R buffer of pH5. Each solution was transferred into the electrolysis cell, and then de-aerated with pure nitrogen. A selected accumulation potential was then applied to the HMDE for a selected preconcentration time period, while the solution was stirred at 400 rpm. At the end of the accumulation period, the stirring was stopped and a 5 s rest period was allowed for the solution to become quiescent. Then, the voltammogram was recorded by scanning the potential toward the negative direction using the selected waveform. All the data were obtained at room temperature.

**Table 1:** Characteristics of the calibration curves of SWAdCS voltammetric determination of pioglitazone HCl

*t_acc_*(s)	Linearity range (M)	Regression equation i_p_ (μA) = a + b C	Correlation coefficient (r)	LOD (M)	LOQ (M)

0	3 × 10^-5^ - 2 × 10^-4^	i_p_ = 5.46 + 1.89 C	0.9959	9.92 × 10^-6^	3.01 × 10^-5^
30	5 × 10^-6^ - 9 × 10^-5^	i_p_ = 0.55 + 6.51 C	0.9983	1.04 × 10^-6^	3.16 × 10^-6^
60	2 × 10^-6^ - 4 × 10^-5^	i_p_ = 1.64 + 6.53 C	0.9968	6.37 ×10^-7^	1.93 × 10^-6^
100	7 × 10^-7^ - 8 × 10^-6^	i_p_ = -0.15 + 5.05 C	0.9981	1.60 ×10^-7^	4.86 × 10^-7^
150	2 × 10^-7^ - 3 × 10^-6^	i_p_ = -0.02 + 7.54 C	0.9967	5.82 ×10^-8^	1.76 × 10^-7^
300	4 × 10^-8^ - 6 × 10^-7^	i_p_ = 5.56 + 3.23 C	0.9957	8.08 ×10^-9^	2.45 × 10^-8^

To study the accuracy and precision of the proposed procedure for the determination of the drug in bulk form, tablets and biological fluids samples; recovery experiments were carried out, by the calibration curve.

**Analysis of tablets.** An accurately weighed amount of ten powdered tablets equivalent to 10.0 mg of the drug, was transferred into a 50 ml volumetric flask, then methanol was added to the flask and completed to the mark. The flask with its contents were sonicated for 15 min and filtered. The desired concentrations of the drug were obtained by accurate dilution with methanol and used as working standard solution. An aliquot volume of this solution was transferred into a 25 ml volume calibrated flask then made up to the volume with the supporting electrolyte.

**Analysis of spiked urine and serum.** An aliquot of standard methanolic solution of pioglitazone HCl containing 100 μg was added to 1 ml of serum or urine sample in a 15 ml centrifuge tube and mixed for 1 min. 0.2 ml of disodium tetraborate solution (0.02 M) was added and the tube was vortexed for 1 min. 5 ml of dichloromethane was added and the tube was shaken for 10 min. The tube was then centrifuged at 3000 rpm for 10 min at room temperature. The resulting organic layer was removed and the extraction was repeated twice with 5 ml of dichloromethane. The combined extracts were evaporated to dryness at room temperature, and the residue was dissolved in 1 ml of methanol. The solution was transferred into a 10 ml volumetric flask, completed to volume with methanol, and subsequently analyzed according to the recommended in the general analytical procedure. A blank experiment was carried out adopting the above procedure.

**Pharmacokinetic study.** The study was performed on one healthy female volunteer. The volunteer fasted overnight before oral administration and for 4 h after the dosing. Five ml of venous blood sample was collected 0.5 h before dosing to serve as blank, and samples were collected in appropriately labeled lithium-heparin tubes at 0.5, 1, 1.5, 2, 2.5, 3, 5, 7, 14 and 24 h following drug administration. The blood samples were centrifuged immediately at 5000 rpm for 15 min, and plasma was rapidly collected and stored at -20°C until assayed. Each plasma sample was assayed by using the proposed square-wave adsorptive cathodic stripping voltammetric procedure, and the values provide the plasma concentration at the time period of collection of the blood sample. The pharmacokinetic parameters were evaluated from the plasma concentration-time profile.

**Spectral study.** A preliminary study for the spectra (Absorbance - wavelength λ) of 5 × 10^-5^ M pioglitazone HCl in B-R buffers pH2-11 was carried out. The spectra exhibited two bands at 221 and 270 nm within the entire pH range. The maximum absorbance of the first peak increased slightly while that of the second peak decreased practically with the same rate upon the increase of pH. From the S and Z shaped absorbance-pH curves for the first (λ=221 nm) and second (λ=270 nm) bands, respectively, the p*K*_a_ of pioglitazone HCl was estimated and found to equal 5.8 and 6.1, respectively, with a mean p*K*_a_ value of 5.95.

## RESULTS AND DISCUSSION

### Effect of type and pH of the supporting electrolyte

**Figure 2:**
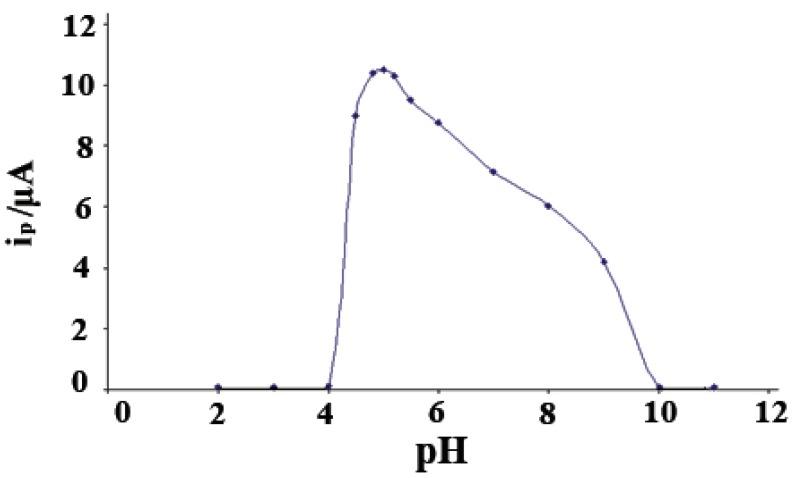
Influence of pH (B–R buffers) on the SWAdCS voltammetric peak current (*i*_p_) of 5 × 10^-5^ M pioglitazone HCl; frequency *f*=120 Hz, scan increment Δ*E*_s_=10 mV and pulse-amplitude *E*_sw_=25 mV, with *t*_acc_=30 s at –1.5 V.

**Figure 3:**
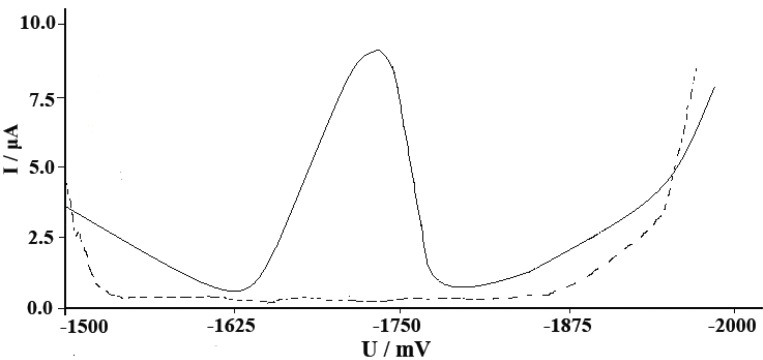
SWAdCS voltammetry of (a) 5 × 10^-5^ M pioglitazone HCl in B–R buffer of pH5 (—), (b) B–R buffer of pH5 (---); frequency *f*=120 Hz, scan increment Δ*E*_s_=10 mV, pulse-amplitude *E*_sw_=25 mV and *t*_acc_=30 s at *E*_acc_ = –1.5 V.

The effect of pH’s was studied on the SWAdCS voltammetric response for 5 × 10^-5^ M pioglitazone HCl and was examined in B-R buffers over the pH range 2-11 with pre-concentration (*t*_acc_=30 s). The voltammograms exhibited a single well-defined four-electron irreversible cathodic peak over the pH range (4.5-8). This peak may be attributed to the hydrogenation of two C=O double bonds via the consumption of four-electrons per drug molecule. The peak potential shifted to more negative values upon pH increase of the medium denoting that protons are involved in the electrode reaction process and that the proton-transfer reaction preceedes the electrode process properly (41-43). As shown in Fig. 2 the peak current intensities (*i*_p_) recorded at different pH values following pre-concentration for 30 s. A much higher peak current intensity was achieved in B-R buffers of pH5. Other supporting electrolytes such as acetate buffer pH(4.5-5.5), sodium sulfate, sodium nitrate, sodium perchlorate and potassium chloride were also tested but the peak current intensity was less developed compared to that obtained in B-R buffer of pH5. Therefore, B-R buffer of pH5 was used as a supporting electrolyte in the rest of the present work. Fig. 3 shows SWAdCS voltammetry of 5 × 10^-5^ M pioglitazone HCl in B-R buffer of pH5.

### Optimization of the proposed analytical procedure

**Figure 4:**
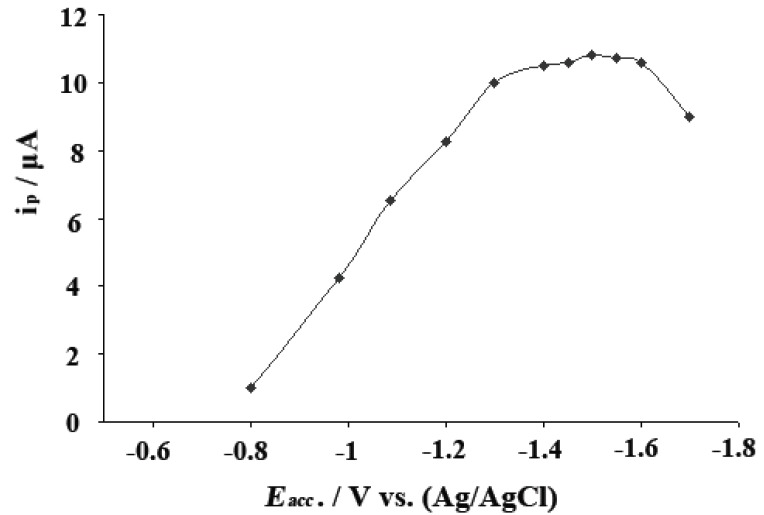
Effect of accumulation potential (*E*_acc_) on the SWAdCS voltammetric peak current (*i*_p_) of 5 × 10^-5^ M pioglitazone HCl in B–R buffer of pH5; frequency *f*=120 Hz, scan increment Δ*E*_s_=10 mV and pulse-amplitude *E*_sw_=25 mV, following preconcentration for *t_acc_*= 30 s.

The SWAdCS voltammetric response markedly depends on the parameters of the excitement signal. In order to reach a maximum developed SWAdCS voltammetric peak current, the optimum instrumental conditions (frequency *f*, scan increment Δ*E*_s_ and pulse amplitude *E*_sw_) were studied for 5 × 10^-5^ M pioglitazone HCl in B-R buffer of pH5, following preconcentration for 30 s. At a scan increment of 10 mV and a pulse-amplitude of 25 mV, the peak current intensity increased linearly over the frequency range (40-120) Hz, following the relationship: *i*_p_ (μA)=0.93 *f* (Hz) + 0.08 (*r*=0.996 and *n*=6). At a frequency of 120 Hz and a pulse-amplitude of 25 mV, the peak current intensity increased linearly with the scan increment up to 10 mV, following the relationship: *i*_p_ (μA)=1.14 Δ*E*_s_ (mV) + 0.96 (*r*=0.997 and *n*=4). Also, at *f*=120 Hz and Δ*E*_s_=10 mV, the peak current increased linearly with the increase of the pulse-amplitude from 15 to 50 mV, however the sharper peak was obtained at 25 mV. Therefore, the optimal instrumental operational conditions of the proposed square-wave procedure can be concluded as: frequency *f*=120 Hz, scan increment Δ*E*_s_=10 mV and pulse amplitude *E*_sw_=25 mV. On the other side, the effect of varying accumulation potential (*E*_acc_) from -0.8 to -1.7 V on the peak current intensity of the SWAdCS voltammogram of 5 × 10^-5^ M pioglitazone HCl in B-R buffer of pH5, following preconcentration for 30 s was evaluated in (Fig. 4). A maximum developed peak current was achieved over the potential range of -1.3 to -1.6 V. The observed gradual decrease in peak current intensity may be attributed to the consequence of desorption of the drug at higher or lower potential values. Hence, an accumulation potential of -1.5 V (versus Ag/AgCl/KCl_sat_) was chosen throughout the present study.

### Effect of accumulation time

**Figure 5:**
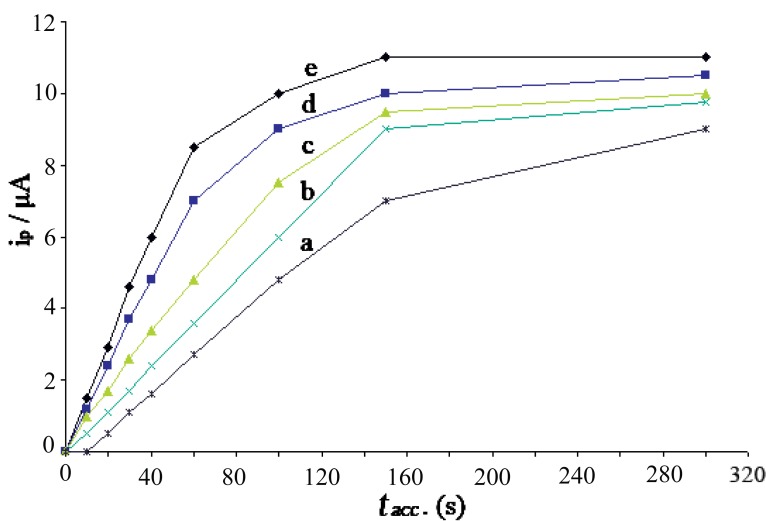
Peak current vs. accumulation time in the presence of B–R buffer of pH5, accumulation potential –1.5 V for: (a) 1 × 10^-6^ M; (b) 3 × 10^-6^ M; (c) 5 × 10^-6^ M; (d) 7 × 10^-6^ M; (e) 9 × 10^-6^ M of pioglitazone HCl.

The SWAdCS voltammogram of 1 × 10^-6^, 3 × 10^-6^, 5 × 10^-6^, 7 × 10^-6^ and 9 × 10^-6^ M pioglitazone HCl were recorded under the optimal operational conditions at increased accumulation time. Fig. 5 shows the effect of different pre-concentration time for the above concentrations of pioglitazone HCl in the presence of B-R buffer of pH5, and accumulation potential of -1.5 V. Straight lines with slopes 0.05 and 0.06 μA s^-1^ were obtained for 1 × 10^-6^ and 3 × 10^-6^ M pioglitazone HCl, respectively where the straight lines break at *t_acc_*=150 s. However at higher concentrations, the straight lines break at *t_acc_*=100 s for 5 × 10^-6^ M pioglitazone HCl and at *t_acc_*=60 s for 7 × 10^-6^ and 9 × 10^-6^ M pioglitazone HCl, respectively. The breaks at certain stirring accumulation times mean that surface coverage was attained. The slope of the straight lines are 0.07, 0.12 and 0.14 μA s^-1^ for 5 × 10^-6^, 7 × 10^-6^ and 9 × 10^-6^ M pioglitazone HCl, respectively and the increase in the slope is due to the increase in the concentration. The collected data are summarized in Table 2.

**Table 2:** Characteristic of current–time curves established using different concentrations of pioglitazone HCl in B–R buffer of pH5

Pioglitazone HCl (M)	Regression equation i_p_ (μA) = a + b C	Correlation coefficient (r)	Linearity range (s)	*δ* (a)	*δ* (b)

1 × 10^-6^	i_p_ = -0.57 + 0.05 C	0.9962	20 - 150	0.175	0.0016
3 × 10^-6^	i_p_ = -0.08 + 0.06 C	0.9981	10 - 150	0.122	0.002
5 × 10^-6^	i_p_ = 0.29 + 0.07 C	0.9976	10 - 100	0.133	0.003
7 × 10^-6^	i_p_ = 0.05 + 0.12 C	0.9963	0 - 60	0.170	0.005
9 × 10^-6^	i_p_ = 0.09 + 0.14 C	0.9987	0 - 60	0.120	0.004

*δ*(a), standard deviation of intercept; *δ*(b), standard deviation of slope.

### Effect of concentration

The SWAdCS voltammetry of series of solutions containing different concentrations of pioglitazone HCl in the range cited in (Table 1) were recorded under optimal operational conditions, *f*=120 Hz, scan increment Δ*E*_s_=10 mV, pulse-amplitude *E*_sw_=25 mV and accumulation potential *E_acc_*=-1.5V at increased accumulation time. The peak current intensity showed a linear relationship with the accumulation time up to 100, 150 and 300 s for the concentration levels of 7 × 10^-7^, 2 × 10^-7^ and 4 × 10^-8^ M of pioglitazone HCl, respectively. This means that the optimal accumulation time period should be chosen according to the concentration level of pioglitazone HCl in the investigated sample (i.e. the higher the concentration, the shorter the accumulation time is). Table 1 illustrates the characteristics of the calibration curves of SWAdCS voltammetric determination of pioglitazone HCl.

### Adsorptive character of the drug

**Figure 6:**
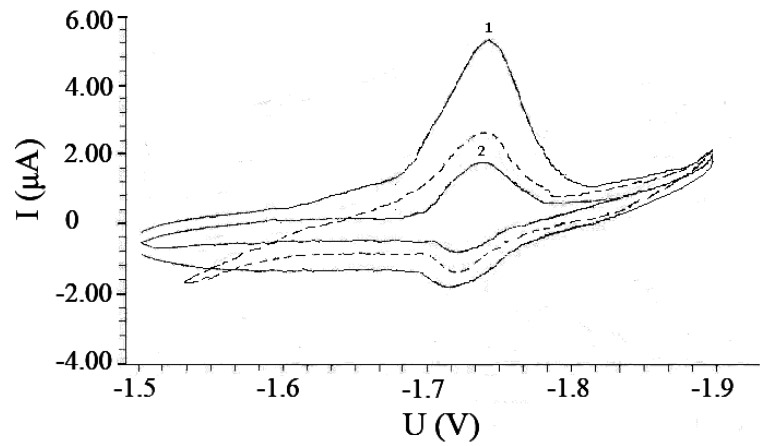
Cyclic voltammograms of 5 × 10^-4^ M pioglitazone HCl in B–R buffer of pH5; without preconcentration (*t*_acc_=0 s) (dashed curve), following preconcentration for 30 s (curve 1) and its repetitive cycle at the same mercury drop (curve 2), Scan rate *ν*=100 mV s^-1^ and *E*_acc_ =-1.5 V.

Fig. 6 shows the cyclic voltammograms of 5 × 10^-4^ M pioglitazone HCl in B-R buffer of pH5, scan rate *v*=100 mV s^-1^ recorded without pre-concentration, *t*_acc_=0 s (dashed curve), following pre-concentration for 30 s (curve 1) and then its repetitive cycle at the same mercury drop (curve 2). A more developed peak current was achieved after pre-concentration of the drug onto the electrode surface, whereas the second cycle at the same mercury drop exhibited lower peak current intensity which may be due to the desorption of drug species out of the mercury electrode surface. This behavior indicated the interfacial adsorptive character of the drug onto the mercury electrode surface.

### Analytical applications

**Analysis of tablets.** The developed SWAdCS voltammetric procedure was applied (*t*_acc_=60 s) for the determination of pioglitazone HCl in its pharmaceutical formulations (Actos tablets 15 and 30 mg pioglitazone HCl) without the necessity for any extraction steps. Recoveries of pioglitazone HCl in both dosage forms, based on the average of three replicate measurements, are illustrated in Table 3. The results were statistically compared with those obtained by the published spectrophotometric method (28). Values of Student’s *t*-value, Variance ratio, are also included. Since the calculated Variance ratio did not exceed the theoretical one, there was no significant difference between the proposed and puplished methods with respect to accuracy and precision (44).

**Table 3:** Analysis of pioglitazone HCl in its tablets by the proposed SWAdCS voltammetry (in B–R buffer of pH 5 and *t_acc_* = 60 s) and the published spectrophotometric methods (28)

Preparatin	Concentration taken (M)	Found (%)	
		Proposed method^b^	Published method (28)

Actos tablets^a^	3 × 10^-5^	100.4	
(15 mg pioglitazone HCl/tablet)	1 × 10^-5^	98.3	
	7 × 10^-6^	99.8	
	5 × 10^-6^	101.9	
	3 × 10^-6^	101.2	
Mean ± S.D.		100.3 ± 1.38	100.6 ± 1.44^c^
Student’s t-value		0.29 (2.447)^d^	
Variance ratio		1.09 (6.94)^e^	
Actos tablets^a^	3 × 10^-5^	100.0	
(30 mg pioglitazone HCl/tablet)	1 × 10^-5^	101.0	
	7 × 10^-6^	101.5	
	5 × 10^-6^	99.2	
	3 × 10^-6^	99.0	
Mean ± S.D.		100.1 ± 1.09	99.7 ± 1.53^c^
Student’s t-value		0.43 (2.447)^d^	
Variance ratio		1.97 (6.94)^e^	

a Products of Takeda chemical industries Ltd.,Osaka.Japan;

b Each result is the avarage of three separate determinations;

c Mean ± S.D. for three different concentrations;

d Tabulated t-value at confidence level 95% (44);

e Tabulated F-value at confidence level 95% (44).

**Analysis of spiked urine and serum.** The high sensitivity attained by the proposed method allows the determination of pioglitazone HCl in biological fluids. Pioglitazone HCl is rapidly absorbed after oral administration. Peak plasma concentrations are obtained within 2 hours and bioavailability exceeds 80%. Pioglitazone HCl is more than 99% bound to plasma proteins. It is extensively metabolised by cytochrome P450 isoenzymes CYP3A4 and CYP2C9 to both active and inactive metabolites. It is excreted in urine and faeces and has a plasma half-life of up to 7 hours. The active metabolites have a half-life of up to 24 hours (45).

As a consequence, the proposed method appears to be convenient for the therapeutic drug monitoring in urine and serum. In addition, the SWAdCS voltammetry, requiring smaller volumes of samples, may be valuable for routine drug screening of patients under treatment. The extraction procedure for biological fluids was performed by using dichloromethane, as reported by Kolte et.al (46). Table 4 and 5 show the performance data and the results for determination of pioglitazone HCl in urine and serum, respectively. The results show that pioglitazone HCl can be determined in spiked serum and urine over the concentration range 6 × 10^-8^- 4 × 10^-7^ M.

**Table 4:** Performance data of piogletazone HCl by the proposed SWAdCS voltammetry (in B–R buffer of pH5 and *t_acc_* =300 s) in serum and urine

Sample	Linearity range (M)	Regression equation i_p_ (μA) = a + b C	Correlation coefficient (r)^a^	LOD (M)	LOQ (M)

Urine	6 × 10^-8^ – 4 × 10^-7^	i_p_ = 4.04 + 2.89 C	0.9983	1.62 × 10^-8^	4.91 × 10^-8^
Serum	6 × 10^-8^ – 4 × 10^-7^	i_p_ = 3.94 + 2.45 C	0.9981	1.64 × 10^-8^	4.98 × 10^-8^

a 6 data points.

**Table 5:** Determination of pioglitazone HCl in spiked serum and urine by the proposed SWAdCS voltammetry, *t_acc_*=300 s

Urine		Serum	
Concentration taken (M)	Found (%)	Concentration taken (M)	Found (%)

6 × 10^-8^	100.2	6 × 10^-8^	99.3
7 × 10^-8^	98.3	7 × 10^-8^	102.0
8 × 10^-8^	100.4	9 × 10^-8^	98.0
1 × 10^-7^	100.6	1 × 10^-7^	100.4
3 × 10^-7^	101.0	4 × 10^-7^	99.2
Mean ± S.D.	100.1 ± 1.05	Mean ± S.D.	99.9 ± 1.48

**Figure 7:**
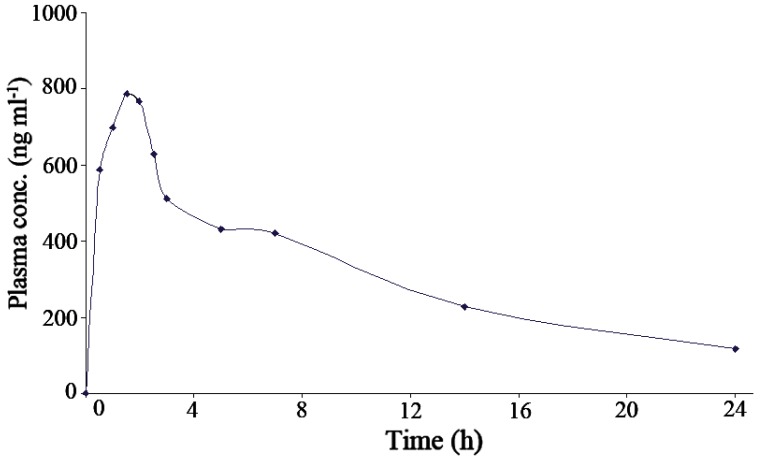
Plasma concentration-time profile for healthy female volunteer after oral administration of 30 mg pioglitazone HCl tablet.

**Pharmacokinetic application.** In a trial to prove the utility of the proposed SWAdCS voltammetric procedure in clinical analysis, the procedure was applied to determination of the pharmacokinetic parameters of pioglitazone HCl in blood samples of one healthy female volunteer following an oral administration of a single dose of Actos tablets (30 mg). Fig. 7 shows the plasma concentrations versus time profile. The obtained pharmacokinetic parameters are shown in Table 6. The obtained results were favorably compared with those obtained by reported methods (47-50), which can be considered as an indication for the reliability of the proposed procedure for assay of pioglitazone HCl in real human plasma samples without interference from its metabolites.

**Table 6:** Estimated pharmacokinetic parameters of pioglitazone HCl in plasma of one healthy female volunteer following an oral administration of a single dose of 30 mg Actos tablet

Parameter	Value	Reported value (47-50)

*C*_max_^a^ (ng ml^-1^)	785.8	1010 – 1050
*t*_max_^b^ (h)	1.5	1.5 – 2
*t*_1/2_^c^ (h)	8	8 – 9
*K*_e_^d^ (h^-1^)	0.125	0.125 – 0.111
AUC^e^ (ng h ml^-1^)	8464.2	10890.0 – 10980.0

a The maximum measured plasma concentration of drug;

b Time to reach maximum measured plasma concentration of drug;

c The elimination half-life time;

d The elimination rate constant;

e The area under the curve.

## VALIDATION OF THE ANALYTICAL PROCEDURE

Validation of the procedure for the quantitative assay of pioglitazone HCl was examined via evaluation of the limit of detection (LOD), limit of quantitation (LOQ), repeatability, recovery and robustness. Calibration curves within the concentration levels of 10^-8^ and 10^-4^ M pioglitazone HCl were attempted following different accumulation time periods (0-300 s). The regression equations associated with the calibration plots exhibited good linearity (Table 1) that supported the validation of the proposed procedure for quantitation of pioglitazone HCl. The limits of detection (LOD) and quantitation (LOQ) were calculated using the relation *k*(*δ* a)/*b* (51), where *k*=3.3 for LOD and 10 for LOQ, (*δ* a) is the standard deviation of the intercept and *b* is the slope of the calibration curve. Both LOD and LOQ values are shown in Table 1.

The repeatability of the results obtained by means of the proposed SWAdCS voltammetric procedure was examined by performing 15 replicate measurements for 5 × 10^-5^ M pioglitazone HCl following pre-concentration for 30 s. A mean recovery of 99.3 ± 0.77 (n=15) and RSD%<2 were achieved, that indicated high precision of the proposed procedure for assay of the drug.

The selectivity of the optimized procedure for the assay of pioglitazone HCl was examined in the presence of some common excipients (usually present in formulations, e.g. starch, gelatin, lactose, talc and magnesium stearate) and so there is no significant interference from excipients. Accordingly, the proposed procedure can be considered selective.

The robustness (51) of results of a procedure is the ability to remain unaffected by small changes in its operational parameters such as pH and accumulation potential. In the present work, this was examined by studying the effect of small variation of pH(4.8-5.2) and accumulation potential (*E*_acc_=–1.4 to -1.6 V). As shown in Table 7, the recovery values were not significantly affected by these variations and consequently the optimized procedure was reliable for assay of pioglitazone HCl and it could be considered robust (51).

**Table 7:** Influence of small variations of some of the operationl conditions of the proposed procedure on the % Found of 5 × 10^-5^ M pioglitazone HCl; frequency=120 Hz, scan increment=10 mV and pulse-amplitude=25 mV

Variables	Conditions	Mean ± S.D.^a^

pH (B-R buffer)		
4.8	*E_acc_*=-1.5 V	99.4 ± 0.40
5	*t_acc_*=30 s	99.5 ± 0.50
5.2		99.2 ± 0.59
Accumulation potential (*E_acc_*)		
-1.4 V	pH=5	99.3 ± 0.47
-1.5 V	*t_acc_*=30 s	99.5 ± 0.50
-1.6 V		99.4 ± 0.32
Elapsed time (day)		
1	*E_acc_*=-1.5 V	99.5 ± 0.50
2	pH=5	99.4 ± 0.45
3	*t_acc_*=30 s	99.2 ± 0.56
Reproducibility (n=15)	*E_acc_*=-1.5 V	99.3 ± 0.23
	pH=5	
	*t_acc_*=30 s	

a Three different concentrations.

## CONCLUSION

A new highly sensitive, rapid, selective and reproducible SWAdCS voltammetric procedure for the determination of pioglitazone HCl drug in bulk form, pharmaceutical formulations and biological fluids is described. The proposed procedure showed clear advantages, e.g. short period of real time of drug analysis, and a very simple extraction procedure was used for urine and serum samples. Moreover, the proposed method could be applied in clinical laboratories and pharmacokinetic studies because of its very low limits of detection and quantitation.
